# Anti-Inflammatory Activity of a Peptide from Skipjack (*Katsuwonus pelamis*)

**DOI:** 10.3390/md17100582

**Published:** 2019-10-13

**Authors:** Zhi-gao Wang, Xiao-guo Ying, Peng Gao, Chun-li Wang, Yi-fan Wang, Xin-wei Yu, Jing Chen, Bin Wang, Hong-yu Luo

**Affiliations:** 1 Key Laboratory of Health Risk Factors for Seafood of Zhejiang Province, College of Food Science and Pharmacy, Zhejiang Ocean University, Zhoushan 316022, China; wzg940518@163.com (Z.-g.W.); yingxiaoguo@zjou.edu.cn (X.-g.Y.); lingli05250101@163.com (C.-l.W.); wyf284483989@163.com (Y.-f.W.); chenjing1979@126.com (J.C.); wangbin4159@hotmail.com (B.W.); 2 College of Marine Science and Technology, Zhejiang Ocean University, Zhoushan 316022, China; igao@me.com; 3 Key Laboratory of Health Risk Factors for Seafood of Zhejiang Province, Zhoushan Center for Disease Control and Prevention, Zhoushan 316021, China; Xwyu0716@163.com

**Keywords:** skipjack enzymatic peptide, transgenic zebrafish, neutrophil granulocytes, ulcerative colitis, intestinal flora

## Abstract

In this paper, the effect of skipjack (*Katsuwonus pelamis*) enzymatic peptide (SEP), which was prepared and purified from a byproduct of skipjack, on inflammation, ulcerative colitis and the regulation of intestinal flora was studied in a mouse ulcerative colitis model and a transgenic zebrafish inflammation model. The aggregation of transgenic granulocyte neutrophils in zebrafish from a normal environment and from a sterile environment was calculated, and the anti-inflammatory activity of SEP was evaluated. To evaluate the anti-ulcerative colitis activity of SEP, DSS-induced colitis mice were given SEP, salicylazosulfapyridine (SASP), or SASP + SEP. Then, the concentrations of IL-6, IL-10 and TNF-α in the serum were detected, the HE-stained colon tissue was examined by microscopy the species composition and abundance distribution of the intestinal flora was analyzed. The results showed that 500 μg/mL SEP treatment significantly alleviated neutrophil granulocyte aggregation in the zebrafish inflammation model; Diarrhea, hematochezia and body weight loss were alleviated to a certain extent in mice gavaged with SEP and SASP, and the combination of SASP with SEP was the most effective in mice. The damage to villi in the intestine was completely repaired, and the levels of IL-6, IL-10 and TNF-α, which are associated with inflammation, were all reduced. In addition, the proportion of intestinal probiotics or harmless bacteria increased, while that of pathogenic bacteria decreased, and the effect of the combined treatment was the most pronounced. These results show that SEP could relieve inflammation, cure ulcerative colitis, regulate intestinal flora and enhance the therapeutic effect of the clinical drug SASP. This study provides a theoretical basis for the development of SEP as an anti-inflammatory adjuvant therapy and intestinal flora regulator.

## 1. Introduction

The initiation of inflammation, the manifestation of infection or injury, is mediated by resident immune cells via pathogen recognition receptors (PRRs), such as Toll-like receptors (TLRs) [1], leading to the synthesis of soluble mediators such as proinflammatory cytokines (see Glossary), which activate downstream proinflammatory signaling pathways [2]. Concomitantly, the upregulation of cell adhesion molecules on circulating leukocytes and endothelial cells promotes the influx of granulocytes and mononuclear phagocytes from the blood. Upon arrival at a site of inflammation, granulocytes such as neutrophils primarily phagocytose and eliminate tissue debris and microorganisms through distinct intracellular mechanisms, such as the production of reactive oxygen species (ROS), including hydrogen peroxide, superoxide anions, and hydroxyl radicals, and/or extracellular mechanisms via the release of neutrophil extracellular traps (NETS) [3]. The most widely used inflammation treatment approach is to inhibit the synthesis or action of mediators that drive inflammation using drugs including nonsteroidal anti-inflammatory drugs (NSAIDs) and anticytokine therapies, such as anti-tumor necrosis factor (TNF)-α [4].

Ulcerative colitis (UC) is a chronic nonspecific inflammatory disease of the colon and rectum characterized by recurrent abdominal pain, diarrhea, and mucus pus and blood. In recent years, the rate of incidence and prevalence of UC has increased year over year, and the current prevalence rate of UC in China is approximately 11.6/10^6^ [5]. UC leads to an increased incidence of precancerous colorectal cancer lesions, with 9% to 11% of UC patients eventually dying from colon and rectal cancers [6]. Therefore, UC is listed as a modern intractable disease by the WHO and is also a current research hotspot in the field of digestion.

Currently, the etiology of UC remains unclear. Researchers generally believe that the pathogenesis of UC is related to intestinal flora dysbiosis, immune dysfunction, oxidative stress and the involvement of inflammatory mediators [7]. In addition, the structure of the intestinal flora in UC patients displays serious imbalances, such as an increase in the abundance of *Escherichia coli*, *Pseudomonas*, *Salmonella*, *Campylobacter jejuni*, *Clostridium difficile*, *Ruminococcus*, Enterobacteriaceae, Proteobacteria, and *Actinobacteria* and a decrease in the abundance of *Clostridium leptum*, *Firmicutes*, Clostridium IV group and *Bacteroidetes*. Therefore, current study data support the hypothesis that intestinal flora are an initiating and promoting factor in the pathogenesis of UC and play a crucial role in the pathogenesis of UC [8].

The inflammatory response is a defense response mainly produced by living tissues of the vascular system in response to injury factors and characterized by tissue infiltration of white blood cells (granulocytes and macrophages). In zebrafish, the body is transparent in the early stages of development, and neutrophil granulocytes are produced before the fish’s innate immune system forms. In response to specific drugs that induce the production of inflammatory mediators, zebrafish neutrophils produce an immune response and accumulate at the inflammation site. Therefore, the transgenic fluorescent neutrophil zebrafish inflammation model can be used to screen and evaluate anti-inflammatory agents. Animal disease model methods are commonly used in anti-UC experiments, including immunological methods and chemical stimulation methods. Immunological models include the fetal mouse colon implantation model, rat colon bacterial infection model, colonic mucosal tissue sensitization model, immune complex model and the formalin model. However, due to the complexity and poor reproducibility of these experiments, they are rarely used anymore. Chemical stimulation is a common method for the induction of acute UC because of its simple method, short model period, high success rate, low cost and obvious symptoms. Common chemicals used in this method are acetic acid, oxazolone, iodoacetamide, sodium nitrite peroxide and dextran sulfate solution (DSS). DSS-induced UC is a nonspecific ulcerative colitis model with good reproducibility, consistency, simple preparation and similar symptoms and pathological changes to human UC. Therefore, it has become an internationally recognized and widely used model [9]. 

In the screening and efficacy evaluation of anti-inflammatory substances, peptides have gained popularity in recent years. Yin suggested that the combination of TNF-binding peptide and TNFR-blocking peptide can effectively reduce the pathological colon tissue damage in rats with trinitro-benzene-sulfonic acid (TNBS)-induced UC [10]. Jia showed that lactogenic casein glycogen giant peptide (CGMP) can improve oxazolidine-induced UC in mice, confirming that CGMP has good prospects as a nutritional method to regulate ulcerative colitis [11]. Zhang used Caco-2 cells and a DSS-induced colitis mouse model to evaluate the anti-inflammatory activity of 6 peptides isolated from egg white and found that 4 of the peptides had different effects on inflammatory signaling pathways and had anti-inflammatory effects [12]. Popov first found three peptides with anti-inflammatory activity in the skin secretions of tree frogs, among which AC12 (ACFLTRLGTYVC) has the greatest anti-inflammatory activity [13]. In addition, Joshi hydrolyzed gastropod viscera with trypsin, alkaline protease and pepsin. After separating and purifying the products, they found that a nontoxic and low-molecular-weight hexapeptide (Ala-Lys-Gly-Thr-Trp-Lys) had anti-inflammatory effects [14].

The intestinal microbiome has established a symbiotic relationship with the host through long-term evolution and plays an important role in the development and maturation of the host immune system [15]. Balanced intestinal flora is a natural defense barrier to foreign pathogens, and can effectively prevent the invasion of foreign pathogens [16]. The destruction of the intestinal flora structure causes an abnormal immune response, which leads to intestinal tissue damage [17]. Therefore, maintaining the balance of the intestinal flora is helpful for the treatment of UC. Proteobacteria contain many pathogenic strains, such as *E. coli*, *Salmonella*, *Vibrio cholerae*, *Helicobacter pylori* and other well-known strains, as an unstable family of intestinal flora, increase the susceptibility of mice to colitis [18]. Therefore, reducing the abundance of Proteobacteria is helpful to alleviate UC. The colonization of germ-free mice by Bacteroides can correct an underdeveloped immune system [19]. The presence of Bacteroides can help to alleviate inflammation [20]. Enterococcaceae are one of the normal bacterial flora components in humans and animals. Zhang Fen found an *Enterococci faecalis* strain that can reduce cholesterol synthesis [21]. In addition, Zhang showed that *E. faecalis* can be widely used as a kind of probiotic in feed applications and can produce a variety of probiotics in the host [22]. Rikenellaceae prevents enteritis and protects the intestines. Chen et al. found that after giving antibiotics to mice, the percentages of Klebsiella, Rikenellaceae and other bacteria increased significantly, while the percentages of Lactobacillus, Bacteroidales (S24-7) and other bacteria decreased significantly [23]. Ruminaceae can degrade numerous polysaccharides, such as glucan, mannose, resistant starch and simple sugars [24]. In addition, researchers analyzed the intestinal microbial composition of the elderly in long-lived and non-long-lived areas and found that the increase in Ruminococcaceae diversity may be related to human longevity [25].

Based on the preparation of skipjack enzymatic peptides, the objective of this study is to investigate the anti-inflammatory, anti-UC and regulatory the intestinal flora structure activity of SEP and provide theoretical basis for developing marine natural anti-inflammatory drugs or auxiliary treatment food.

## 2. Results and Discussion

### 2.1. Evaluation of the Anti-Inflammatory Activity of SEP in Transgenic Zebrafish

#### 2.1.1. SEP Minimal Toxic Concentration (MTC)

Table 1 shows that SEP was significantly precipitated at concentrations of 1000, 1500 and 2000 μg/mL. No significant abnormalities were observed in the other groups, suggesting that the MTC of SEP is 500 μg/mL. Therefore, 500 μg/mL was selected as the test concentration for the anti-inflammatory activity evaluation.

#### 2.1.2. SEP Anti-Inflammatory Activity Evaluation Results

**Figure 1:**
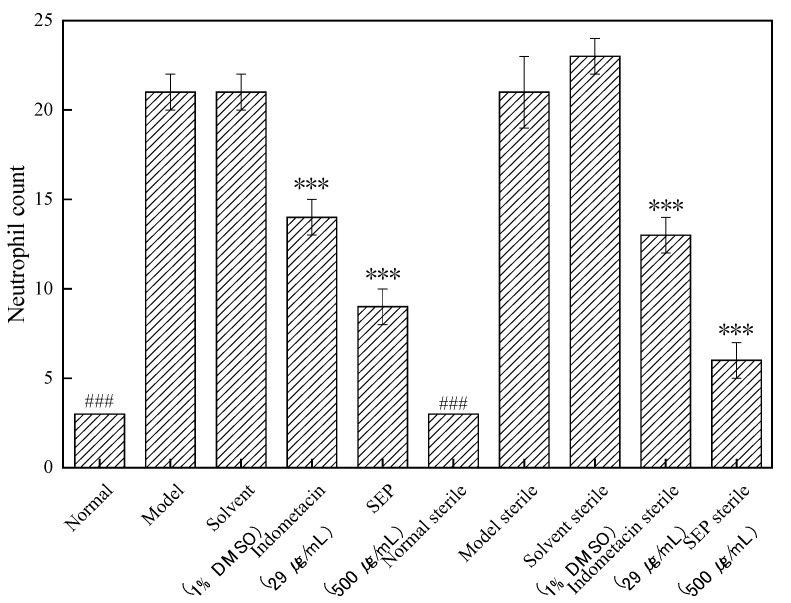
Number of neutrophils in zebrafish in each group. Compared with the model group: ### *p* < 0.001. Compared with the solvent control group: *** *p* < 0.001.

**Figure 2:**
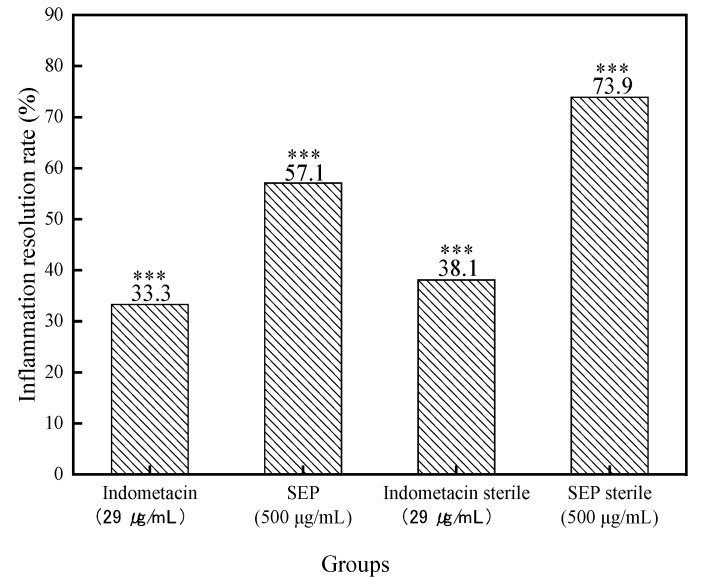
Inflammation abatement effect in each group of zebrafish. Compared with the solvent control group: *** *p* < 0.001.

**Figure 3:**
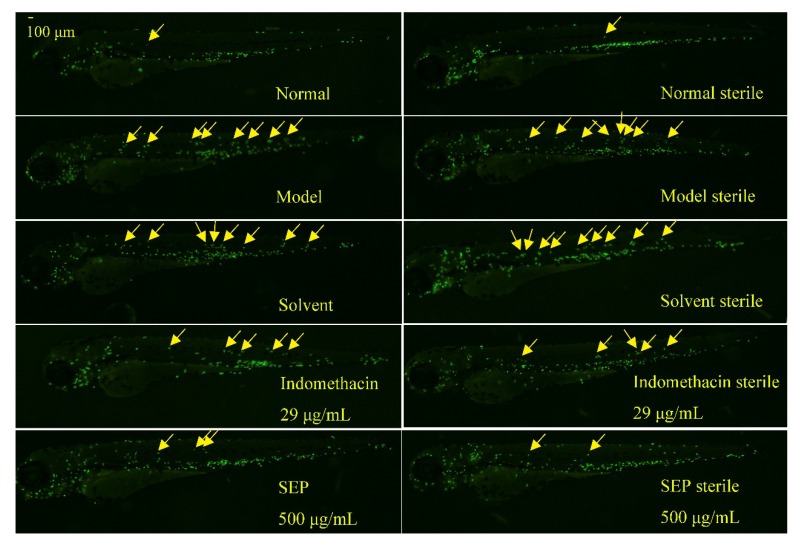
Phenotype diagram of the effect of different treatments on inflammation in zebrafish. Note: the yellow arrows indicate neutrophil in the inflammation site.

The number of neutrophils in the zebrafish of each experimental group is shown in Figure 1, and the rate of inflammation regression is shown in Figure 2. Figure 3 shows the effect of each experimental treatment on zebrafish inflammation.

In sterile and bacteria-bearing conditions, the difference in the number of neutrophils in the inflammation sites of zebrafish was extremely significantly different (*p* < 0.001, Figure 2) between the model group and the normal group, indicating that the copper sulfate-induced zebrafish inflammation model was successfully established. Compared with the solvent control and the copper sulfate, 1% DMSO had no effect on zebrafish inflammation (*p* > 0.05), whether in sterile or bacteria-bearing conditions. However, compared with that in the solvent control group, the number of neutrophils in the indomethacin group (29 μg/mL) was significantly reduced (*p* < 0.001); the inflammation regression rate was 38.1% and 33.3%, respectively. SEP (500 μg/mL) and indomethacin showed the same performance, except that the inflammation regression rate was higher in indomethacin-treated fish (57.1% and 73.9%, respectively), suggesting that SEP and indomethacin have significant anti-inflammatory effects in zebrafish.

### 2.2. Effects of SEP on Body Weight and Disease Activity Index (DAI) Score of Mice with UC

Mice in the model group began to display symptoms of slight hematochezia and diarrhea on the fourth day, which became worse on the sixth, but no symptoms were observed in the normal group. Compared with those in the model group, the levels of blood staining and diarrhea in the packing of the low concentration SEP group were reduced. The symptoms of the high-concentration SEP group were further improved but still present, whereas the symptoms of the SASP and SASP + SEP groups disappeared, and the mice displayed significantly more behavioral activity than the low- and high-concentration SEP group mice. 

**Figure 4:**
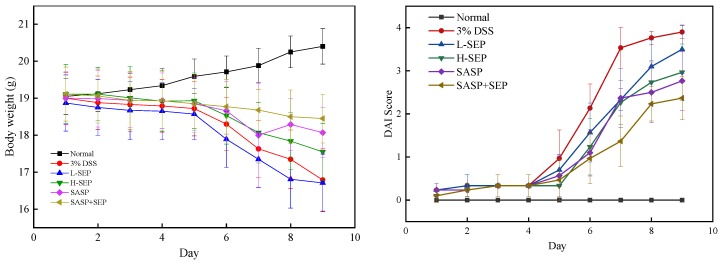
Daily weight change and disease activity index (DAI) score change of mice.

The weight changes and DAI score change of the six groups of mice are shown in Figure 4. Mice in the model group and the low concentration SEP group showed significant weight loss on the sixth day. Mice in the high-concentration SEP group and SASP group began to lose weight slowly by day 7, with the SASP group subsequently regaining weight. Throughout the entire experiment, the weight of mice in the SASP + SEP group decreased slightly and was significantly different from that of mice in the other groups (*p* < 0.05). It has been suggested that SEP can significantly alleviate the weight loss caused by UC. The effect of SEP alone was not as good as that of SASP, but the combination of SEP and SASP had the strongest effect on inhibiting weight loss. 

### 2.3. Effect of SEP on Colonic Tissue Structure in UC Mice

**Figure 5:**
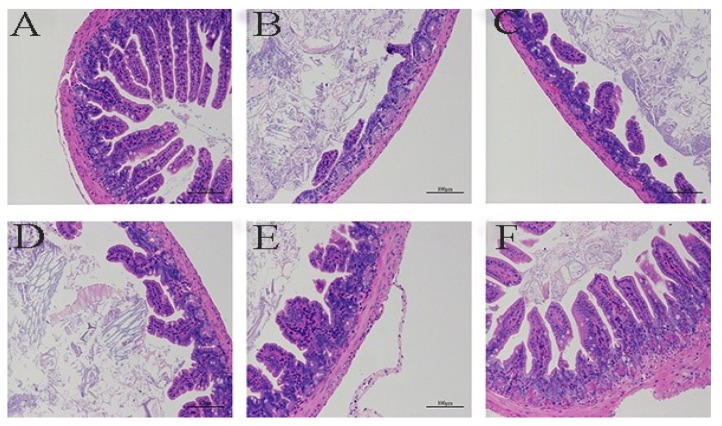
Morphological changes in the colon tissue from mice in each group (HE staining). **A**. normal; **B**. model; **C**. Low-dose SEP; **D**. High-dose SEP; **E**. SASP; and **F**. SASP + SEP.

The HE staining of colon tissue from each group of mice is shown in Figure 5. In the normal group, the colonic mucosa was intact, and the intestinal epithelial cells and glands were arranged neatly (Figure 5A). In the model group, the colon integrity was extremely poor. In these areas, the villus structure was damaged, and the number of intestinal epithelial goblet cells was significantly reduced (Figure 5B). The condition of the colons from the low- and high-concentration SEP mice was better than that of the model mice, with improved villus fracturing and shedding (Figure 5C,D). Intestinal villus rupture and shedding in the SASP group was further improved compared with that in the high-concentration SEP group (Figure 5E). The above symptoms were not present in the SASP + SEP group (Figure 5F). Therefore, SEP has a slowing effect on the progression of DSS-induced intestinal damage. The anti-UC effect of SASP was greater than that of SEP, and the tissue inflammation was greatly reduced. However, the combination of SASP and SEP basically repaired and healed the tissue inflammation in mice, which further suggests that SEP has an auxiliary effect in the treatment of UC.

### 2.4. Effect of SEP on Serum IL-10, IL-6 and TNF-α Levels in Mice with UC

IL-10 is mainly produced by mononuclear macrophages; it has a multidirectional inhibitory effect on biological activity and can inhibit mononuclear macrophage activation and cytokine production. The expression level of IL-10 in patients with UC is significantly reduced, resulting in the increased activation of monocytes and decreased inhibition of the activity of various proinflammatory cytokines (such as IL-6) [26]. IL-6 is a glycoprotein with a molecular weight of 22–28 kDa that has a wide range of proinflammatory effects. Studies have shown that when UC occurs, the IL-6 level is significantly increased [27]. IL-6 interacts with TNF-α, causing a series of inflammatory reactions during the active phase of UC. TNF-α is a non-glycoprotein produced by macrophages, T cells and monocytes in response to stimulation by superantigen or endotoxin. The increase in TNF-α levels is associated with tissue damage, such as apoptosis, inflammation and metabolism, and induces the recruitment of inflammatory cells, thereby increasing the expression of IL-6 and adhesion molecules. TNF-α is an important factor for biological immune defense and internal environment stability, and its expression is related to mucosal injury caused by ulcerative colitis [28]. 

**Figure 6:**
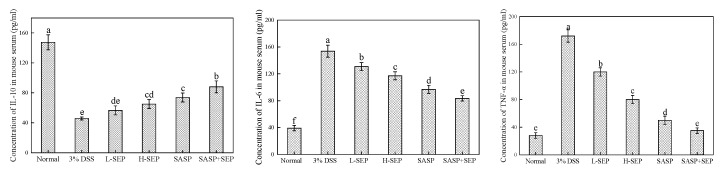
Levels of IL-10, IL-6 and TNF-α in the serum of mice in each group. Note: a–f Values with same letters indicate no significant difference (*p* > 0.05).

The levels of IL-10, IL-6 and TNF-α in the serum of each group of mice are shown in Figure 6. Compared with that in the model group, the IL-10 level in the two SEP groups was significantly increased (*p* < 0.05). The SASP + SEP group showed the highest level of IL-10, which was significantly different from that in the other groups (*p* < 0.01). The IL-6 and TNF-α concentration in the model group was higher than that in the other groups, and the difference was significant. In mice gavaged with different concentrations of SEP, the IL-6 and TNF-α concentrations decreased to different degrees but remained higher than those in the normal group mice. In the SASP group, the expression levels of IL-6 and TNF-α were significantly lower than those in the high-dose SEP group (*p* < 0.01); the reduction was further increased in the SASP + SEP group with a significant difference (*p* < 0.05), indicating that the high dose of SEP had an obvious adjuvant effect on SASP.

### 2.5. Analysis of the Intestinal Flora Structure in Mice

#### 2.5.1. Diversity of the Intestinal Flora in Mice

The number of effective DNA sequences and the diversity of species within a single sample (alpha diversity) were used to analyze the diversity of the intestinal flora in each sample. 

According to the 97% similarity cutoff (reads with sequence similarity greater than 97% were classified into one operational taxonomic unit (OTU) and regarded as one microbial species), the range of OTUs in the six groups of mice was 37–244 (Table 2). The number of intestinal microbial species decreased significantly after DSS induction. After the administration of different doses of SEP and the positive control drug SASP, the number of species increased to different extents. Notably, in mice treated with the combination of SEP and SASP, the diversity of the intestinal flora was close to that of the normal group. These results indicate that SEP has a proliferation-inducing effect on intestinal microbes in mice with UC, which is beneficial to the diversity of the intestinal flora.

The alpha diversity is represented by the observed species index and Chao index, which represent the observed number of OTUs and the estimated number of OTUs in the samples, respectively.

**Figure 7:**
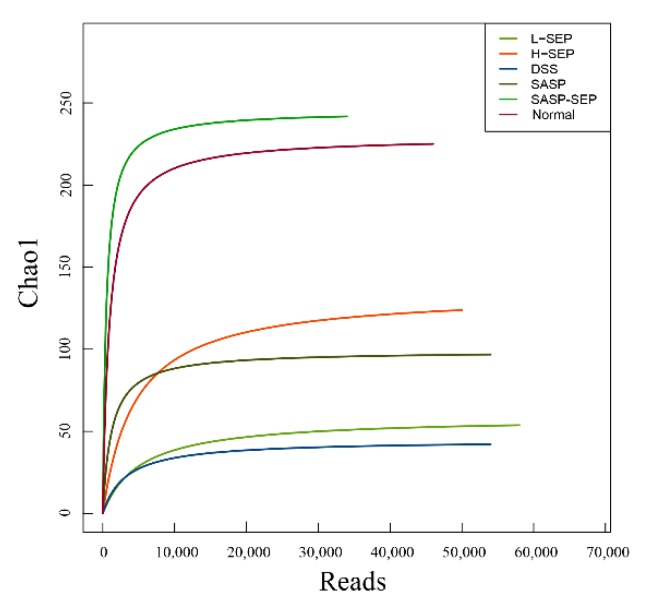
Chao1 dilution curve.

**Figure 8:**
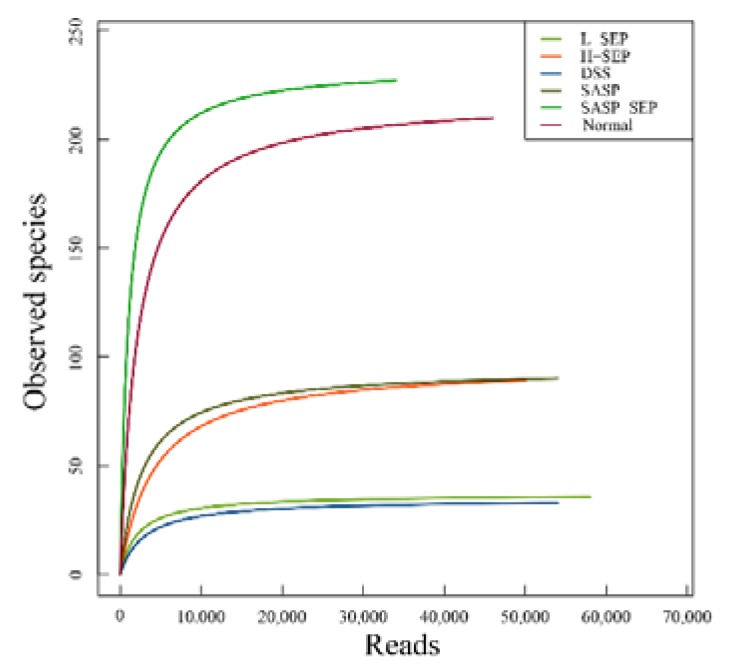
Observed species dilution curve.

The dilution curves of Chao1 and observed species in the six groups are shown in Figure 7 and Figure 8, where the horizontal coordinate represents the number of available bases randomly extracted in the samples, and the vertical coordinate represents the alpha diversity corresponding to the number of reads. When the sequencing quantity of each sample exceeded 10,000, the curves tended to flatten and stopped rising, indicating that no new microorganisms would be amplified even if the sequencing quantity was increased, which suggests that the analysis results of the flora were acceptable.

The observed species and Chao1 indexes in each group were similar, and the biological diversity of the intestinal flora of mice in the normal group and the SASP + SEP group was significantly higher than that of mice in the other groups.

#### 2.5.2. Analysis of Species Abundance

**Figure 9:**
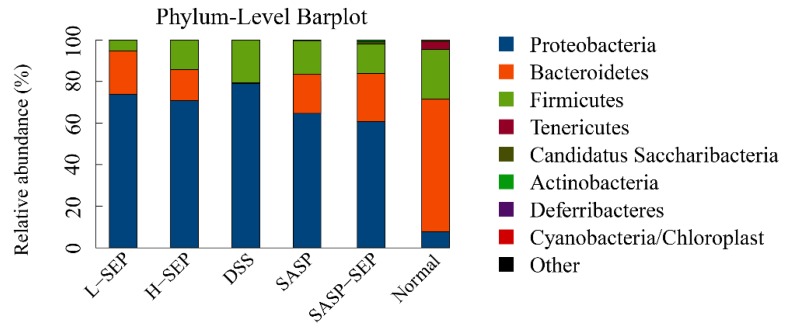
Phylum-level barplot of mouse intestinal flora.

The composition of the intestinal flora of mice in the six groups at the phylum level is shown in Figure 9. The abundance of Proteobacteria in the intestines of the model mice was significantly increased compared with that in the normal group mice, whereas the abundance of Bacteroidetes and Firmicutes was reduced (Table 3).

**Figure 10:**
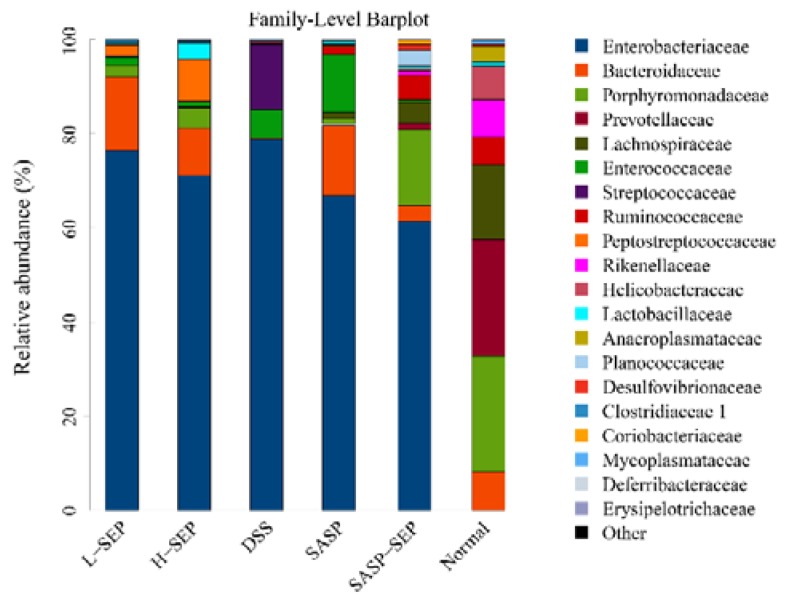
Composition of mouse intestinal flora at the family level.

The results of the species diversity analysis at the family level are shown in Figure 10. Prevotellaceae, Enterococcaceae, Lachnospiraceae and Rikenellaceae were ranked from 1 to 4 in the normal group. Enterobacteriaceae was the most abundant in all experimental groups, while Enterococcaceae, Lachnospiraceae, Prevotellaceae, Ruminococcaceae and Planoconcave all accounted for a certain proportion of the flora in the SASP + SEP group. The second most dominant bacteria in the other experimental groups were all Bacteroidaceae, whereas Streptococcaceae were dominant in the model group. Compared with that in the normal group, the diversity of the intestinal flora of mice in the model and other groups was significantly reduced. However, the SASP + SEP group had more diversity than the other groups, which is consistent with the data in Table 2, Figure 7 and Figure 8.

Compared with the model group, each experimental group showed a reduced abundance of Proteobacteria. Among the groups, the SASP + SEP group showed the greatest effect, decreasing the proportion from 79.12% to 61.23%, although the Proteobacteria abundance in this group remained markedly different from that of the normal group (7.74%). Both SASP and SEP treatment effectively increased the abundance of Bacteroides, and SASP + SEP treatment had the greatest effect, increasing the relative abundance from 0.3357% to 23.87%. These results are consistent with those of previous studies. Firmicutes are associated with metabolism and obesity, and a direct correlation with inflammation has rarely been reported. However, SASP and SEP also had an impact on the Firmicutes.

The use of DSS to induce UC in mice caused fatal damage to the structure of the intestinal flora. SEP and the positive control drug SASP both showed a protective effect on microbial abundance, and the use of SASP with SEP improved its effects. 

At the family level, compared with that of normal mice, the abundance of Enterobacteriaceae and Streptococcaceae in DSS-treated mice was significantly increased. After treatment with SEP and SASP, the abundance of these two families of bacteria was significantly decreased. Zhang showed that the risk of developing rectal cancer is associated with *Streptococcus* in patients [29], suggesting that the intake of SEP and SASP can alleviate rectal cancer. 

In addition, the analysis of the intestinal microflora structure showed that the diversity of the intestinal microflora in mice with DSS-induced UC was disrupted. However, after treatment with SEP and SASP at different concentrations, the diversity of the intestinal flora increased to some extent, suggesting that SEP can improve the intestinal microecology of mice with UC. Zhang found that the abundance of Proteobacteria in the intestinal flora of patients with rectal cancer was significantly higher than that in healthy people, and this increase in Proteobacterial abundance leads to intestinal dysfunction, thereby increasing the risk of disease [29]. Bao also found that the reduction in the abundance of Enterobacteriaceae could alleviate rectal cancer [30]. Our results show that, except for the normal group, the model group and the four experimental groups had the highest relative abundances of Proteobacteria and Enterobacteriaceae in the intestinal tracts of mice, supporting Zhang’s findings. However, compared with that in the model group, the abundance of Proteobacteria and Enterobacteriaceae in the intestinal tracts of mice in all experimental groups was decreased. Notably, in the SEP + SASP group, the relative abundance dropped nearly 18%, indicating that SEP + SASP had a positive effect on maintaining the homeostasis of the intestinal flora. Furthermore, the effect of SEP was increased when it was used in combination with SASP, which is consistent with the results of the anti-inflammatory experiment. 

## 3. Materials and Methods

### 3.1. Materials

#### 3.1.1. Chemical

Skipjack enzymatic peptide (SEP, Leu-Leu-Phe-Thr-Thr-Gln) was isolated and purified by our research group and synthesized by Shanghai Botai Biotechnology Co., Ltd. (Shanghai, China). Kanamycin sulfate, ampicillin sodium, amphotericin B, and polyvinylpyrrolidone iodine complex were purchased from Shanghai Aladdin Biochemical Technology Co., Ltd. (Shanghai, China). NaClO was from Shanghai Ziqin Chemical Co., Ltd. (Shanghai, Chnia). Ethyl alcohol was purchased from Sinopharm Chemical Reagent Co., Ltd. (Shanghai, China). Dimethyl sulfoxide (DMSO) and methyl cellulose were purchased from Sigma-Aldrich (Shanghai, China). DNA rapid extraction kits were purchased from Hangzhou Rongbang Biotechnology Co., Ltd. (Hangzhou, China). Dextran sodium sulfate was purchased from MP Biomedicals (Beijing, China), LLC. Mouse IL-6, IL-10 and TNF-α ELISA kits were purchased from Beyotime Biotech Inc. (Shanghai, China) and sulfasalazine (SASP) was obtained from Zhoushan Hospital of Zhejiang University (Zhoushan, China).

#### 3.1.2. Experimental Animals

Transgenic zebrafish with fluorescent neutrophils were obtained by natural pair mating. The fish were used in experiments at 3 days postfertilization (3 dpf), and all fish were raised in 28 water for fish culture (water quality: To every 1 L of water, 200 mg of instant sea salt was added by reverse osmosis; the conductance was 480–510 μS /cm, the pH was 6.9–7.2, and the rigidity was 53.7–71.6 mg/L CaCO_3_). Breeding management conditions met the international AAALAC certification requirements. Balb/c mice (7 -weeks old, male, weighing 20 ± 2 g) were purchased from Zhejiang experimental animal center (Hangzhou, China). The mice were kept in a specific pathogen-free (SPF) laboratory animal room at a temperature of 24 °C with a 12-h light/dark cycle. Standard chow and water were provided for the animals. All animal experiments were conducted in accordance with relevant regulations of the animal ethics committee of Zhejiang Ocean University.

### 3.2. Equipment

The equipment used in this study include a dissecting microscope (SMZ645, Nikon, Beijing, China); precision electronic balance (CP214, OHAUS, Shanghai, China); electric focus continuous zoom fluorescence microscope (AZ100, Nikon, Shanghai, China); vertical pressure steam sterilizer (LDZX-50KBS, Shanghai Shen An Medical Instrument Factory, Shanghai, China); 6-well plates (Nest Biotech, Shanghai, China); high-speed centrifuge (CR21G, Hitachi Ltd., Beijing, China); water bath (HHS, Shanghai Xunbo Industrial Co., Ltd., Shanghai, China); NanoDrop 2000, and a microplate reader (both from Thermo Fisher Scientific (China) Co., Ltd., Shanghai, China).

### 3.3. Methods

#### 3.3.1. Evaluation of the Anti-Inflammatory Activity of SEP in Transgenic Zebrafish

##### SEP Minimal Toxic Concentration (MTC)

At 3 dpf, transgenic zebrafish with fluorescent neutrophils were randomly placed in six-well plates at 30 tails per well, and water-soluble SEP at concentrations of 31.25, 62.5, 125, 250, 500, 1000, 1500, and 2000 μg/mL was simultaneously administered. Three groups were established: the normal group, the model group and the solvent control group (1% DMSO). After pretreatment for 1 h in a 28 °C incubator, CuSO₄ was used to establish inflammation in the transgenic zebrafish. 2 h later, the number of toxicity-induced zebrafish deaths was observed, and the MTC was determined.

##### Evaluation of the Anti-Inflammatory Activity of SEP

The same batch of fertilized transgenic zebrafish eggs was divided into two equal parts. One part was incubated in normal conditions, and the other was incubated in sterile conditions. The sterile treatment conditions were as follows: Fertilized eggs were incubated in sterile fish water containing 100 μg/mL ampicillin, 5 μg/mL kanamycin, and 250 ng/mL amphotericin B. Well-developed embryos were selected at 6 h and 24 h after fertilization and incubated at 28 °C. The sterile fish water was changed every day up to 3 dpf. All operations were performed under sterile conditions.

At 3 dpf, the zebrafish incubated in normal and sterile conditions were placed in six-well plates, 30 fish per well, and the MTC concentration of SEP was administered. The positive control treatment was 29 μg/mL indomethacin. Therefore, the fish distributed into the following groups: normal group, model group, solvent group (1% DMSO), indomethacin group (29 μg/mL), and SEP group (MTC concentration). After pretreatment for 1 h in a 28 °C incubator, CuSO₄ was used to induce inflammation in the transgenic zebrafish. After 2 h, 10 zebrafish were randomly selected from each group for observation and image capture under a fluorescence microscope. Nikon NIS-Elements D 3.10 advanced image processing software (3.10, Nikon, Shanghai, China) was used to analyze the captured pictures, and the number of neutrophils at the zebrafish inflammation site was calculated (defined as N). The results were expressed as ± SE. The anti-inflammatory effects of SEP on copper sulfate-induced inflammation in the zebrafish were evaluated by statistical analysis of the number of infiltrating neutrophils. The anti-inflammatory efficacy formula was as follows:

$$\mathrm{Inflammation}\;\mathrm{regression}\;\mathrm{rate}\;(\%)\;=\left[\frac{N(\mathrm{Solvent}\;\mathrm{control})-N(\mathrm{Test}\;\mathrm{group})}{N(\mathrm{Solvent}\;\mathrm{control})}\right]\;\times \;100\%$$

Statistical analyses were performed using analysis of variance and Dunnett’s t-test, with *p* < 0.05 considered indicative of significant differences.

#### 3.3.2. Evaluation of the Anti-UC Activity in Mice

**Figure 11:**
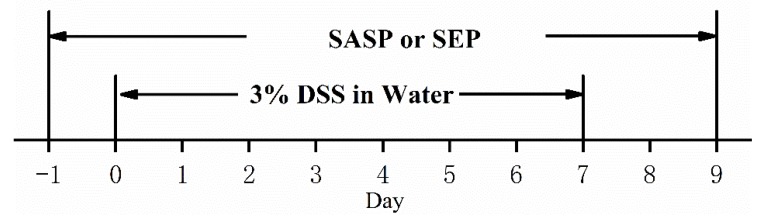
DSS-induced UC mouse model method.

After 7 days of adaptive feeding, the mice were randomly divided into 6 groups, with 10 mice in each group. Mice received a normal diet, and every group of mice was gavaged with 0.1 mL of the appropriate treatment at 18:00 every day. The specific grouping, dosing schedule and modeling method are shown in Table 4 and Figure 11 [31].

During the experiment, the posture of each group of mice was observed, and the body weight of each group of mice was measured (each mouse was accurately weighed once a day). At noon on the 10th day of administration, the mice were subjected to blood collection using the eyeball method (fasting for 12 h and water for 1 h before blood collection). After the eyeballs were removed, the blood was immediately collected into an Eppendorf tube and placed on ice. After 45 min, the blood was centrifuged at 4000 r/min for 10 min at 4 °C, and the supernatant was taken. The serum levels of IL-6, IL-10 and TNF-α were detected by enzyme-linked immunosorbent assay (ELISA) and three parallel tests are required for each sample. After blood collection, the mice were sacrificed by the dislocation method. A DSS-treated colon and a normal colon were taken for hematoxylin and eosin (HE) staining [32].

#### 3.3.3. Extraction and Analysis of DNA from the Gut Flora of Mice

After blood collection, the mice were sacrificed by the dislocation method. The contents of the colon were collected, and the intestinal flora DNA was extracted, and the concentration was detected by NanoDrop 2000. The DNA was extracted samples with the highest concentration for 16S rRNA sequencing. The data were statistically analyzed, and the sequencing work was completed by Shanghai Ruiyi Biotechnology Co., Ltd. (Shanghai, China).

### 3.4. Statistical Analysis

Analyses were performed using SPSS Statistics, version 17.0 (IBM SPSS Inc., Chicago, IL, USA). The data was tested for normal distribution by Shapiro-Wilk test. All the continuous variables were expressed as mean ± standard deviation and median (interquartile range) and were analysed using the Indepedents t-test and Mann Whitney U test, respectively the significant differences were determined at *p* < 0.05.

## 4. Conclusions

In this study, the physiological activity SEP was investigated based on the transgenic zebrafish model and the UC mouse model. The results showed the effect of anti-inflammatory, anti-UC and regulating the structure of intestinal flora of SEP. These results suggest that SEP has the potential to be developed as a natural marine anti-inflammatory drug. At the same time, the combination effect of SEP and SASP is better, which also suggests that SEP has the potential to develop compatible drugs for SASP and health food for adjuvant treatment of inflammation. So far, the anti-inflammatory activity of SEP has been basically determined. The next step is to study the anti-inflammatory mechanism of SEP with cell model and determine the signaling pathway of its efficacy through protein and gene expression.

## Figures and Tables

**Table 1:** Summary of zebrafish toxicity symptoms in each group (*n* = 30).

Groups	Experimental Concentration (μg/mL)	Death Number	Death Rate (%)	Toxicity Performance
Normal	-	0	0	-
Model	-	0	0	-
Solvent control (1% DMSO)	-	0	0	-
SEP	31.25	0	0	-
	62.5	0	0	-
	125	0	0	-
	250	0	0	-
	500	0	0	-
	1000	-	-	precipitated
	1500	-	-	precipitated
	2000	-	-	precipitated

**Table 2:** Sequence statistics.

Group	Effective Sequence Number	Total Number of Bases (bp)	Average Length (bp)	OTUs
Low-dose SEP	60,532	25,733,194	425	42
High-dose SEP	55,524	23,536,705	423	99
DSS	57,059	24,402,954	427	37
SASP	61,383	26,198,618	426	96
SASP + SEP	56,461	23,894,720	423	223
Normal	56,461	18,667,293	416	244

**Table 3:** Main colony composition of mice at the phylum level.

	Low-dose SEP	High-dose SEP	DSS	SASP	SASP + SEP	Normal
Proteobacteria	74.65 (%)	70.52 (%)	79.12 (%)	64.89 (%)	61.23 (%)	7.74 (%)
Bacteroidetes	18.31 (%)	10.12 (%)	0.3357 (%)	13.96 (%)	23.87 (%)	63.88 (%)
Firmicutes	6.28 (%)	15.86 (%)	20.54 (%)	12.14 (%)	11.78 (%)	14.18 (%)

**Table 4:** Mouse grouping and dosing regimen.

Groups	Number of Mice	Induction Drug	Gavage Drugs
Normal	10	Water	Saline
Model	10	3% DSS	Saline
Low-dose SEP	10	3% DSS	1 g SEP /100 mL
High-dose SEP	10	3% DSS	3 g SEP /100 mL
SASP	10	3% DSS	2 g SASP /100 mL
SASP + SEP	10	3% DSS	(2 g SASP + 3 g SEP)/100 mL

Note: SASP was used as a positive control drug for the clinical treatment of UC.
